# A phase 3 study of nivolumab in previously treated advanced gastric or gastroesophageal junction cancer (ATTRACTION-2): 2-year update data

**DOI:** 10.1007/s10120-019-01034-7

**Published:** 2019-12-20

**Authors:** Li-Tzong Chen, Taroh Satoh, Min-Hee Ryu, Yee Chao, Ken Kato, Hyun Cheol Chung, Jen-Shi Chen, Kei Muro, Won Ki Kang, Kun-Huei Yeh, Takaki Yoshikawa, Sang Cheul Oh, Li-Yuan Bai, Takao Tamura, Keun-Wook Lee, Yasuo Hamamoto, Jong Gwang Kim, Keisho Chin, Do-Youn Oh, Keiko Minashi, Jae Yong Cho, Masahiro Tsuda, Hiroki Sameshima, Yoon-Koo Kang, Narikazu Boku

**Affiliations:** 1 National Institute of Cancer Research, National Health Research Institutes, National Cheng Kung University, Tainan, Taiwan; 2 Department of Internal Medicine, National Cheng Kung University Hospital, National Cheng Kung University, Tainan, Taiwan; 3 Frontier Science for Cancer and Chemotherapy, Osaka University Graduate School of Medicine, Suita, Japan; 4 Department of Oncology, Asan Medical Center, University of Ulsan College of Medicine, Seoul, South Korea; 5 Department of Oncology, Taipei Veterans General Hospital, Taipei, Taiwan; 6 Division of Gastrointestinal Medical Oncology, National Cancer Center Hospital, 5-1-1, Tsukiji, Chuo-ku, Tokyo, 104-0045 Japan; 7 Division of Medical Oncology, Yonsei Cancer Center, Song-Dang Institute for Cancer Research, Yonsei University College of Medicine, Yonsei University Health System, Seoul, South Korea; 8 Division of Hematology and Oncology, Department of Internal Medicine, Linkou Chang Gung Memorial Hospital, Chang Gung University, Taoyuan, Taiwan; 9 Department of Clinical Oncology, Aichi Cancer Center Hospital, Nagoya, Japan; 10 Division of Hematology-Oncology, Department of Medicine, Samsung Medical Center, Sungkyunkwan University School of Medicine, Seoul, South Korea; 11 National Taiwan University Cancer Center, Taipei, Taiwan; 12 Department of Oncology, National Taiwan University Hospital, National Taiwan University College of Medicine, Taipei, Taiwan; 13 Department of Gastrointestinal Surgery, Kanagawa Cancer Center, Yokohama, Japan; 14 Division of Hematology and Oncology, Department of Internal Medicine, College of Medicine, Korea University, Seoul, South Korea; 15 Division of Hematology and Oncology, Department of Internal Medicine, China Medical University Hospital, China Medical University, Taichung, Taiwan; 16 Department of Medical Oncology, Faculty of Medicine, Kindai University, Osakasayama, Japan; 17 Division of Hematology and Oncology, Department of Internal Medicine, Seoul National University Bundang Hospital, Seoul National University College of Medicine, Seongnam, South Korea; 18 Keio Cancer Center, Keio University School of Medicine, Tokyo, Japan; 19 Kyungpook National University School of Medicine, Daegu, South Korea; 20 Department of Gastroenterology, Cancer Institute Hospital of the Japanese Foundation for Cancer Research, Tokyo, Japan; 21 Department of Internal Medicine, Seoul National University Hospital, Seoul, South Korea; 22 Clinical Trial Promotion Department, Chiba Cancer Center, Chiba, Japan; 23 Department of Medical Oncology, Gangnam Severance Hospital, Yonsei University College of Medicine, Seoul, South Korea; 24 Department of Gastroenterological Oncology, Hyogo Cancer Center, Akashi, Japan; 25 Medical Oncology, Medical Affairs, Ono Pharmaceutical Co., Ltd., Osaka, Japan; 26 Present Address: Department of Gastric Surgery, National Cancer Center Hospital, Tokyo, Japan; 27 Present Address: Department of Medical Oncology, Kindai University Nara Hospital, Ikoma, Japan

**Keywords:** Gastric cancer, Gastroesophageal junction cancer, Long-term, Nivolumab, Placebo

## Abstract

**Background:**

Nivolumab showed improvement in overall survival (OS) in ATTRACTION-2, the first phase 3 study in patients with gastric/gastroesophageal junction (G/GEJ) cancer treated with ≥ 2 chemotherapy regimens. The 2-year follow-up results of ATTRACTION-2 are presented herein.

**Methods:**

ATTRACTION-2 was a randomized, double-blind, placebo-controlled, phase 3 trial (49 sites; Japan, South Korea, and Taiwan). The median (min–max) follow-up period was 27.3 (24.1–36.3) months. The primary endpoint was OS. A subanalysis of OS was performed based on best overall response and tumor-programmed death ligand-1 (PD-L1) expression status.

**Results:**

Overall, 493 of 601 screened patients were randomized (2:1) to receive nivolumab (330) or placebo (163). OS (median [95% confidence interval; CI]) was significantly longer in the nivolumab group (5.26 [4.60–6.37] vs 4.14 [3.42–4.86] months in placebo group) at the 2-year follow-up (hazard ratio [95% CI], 0.62 [0.51–0.76]; *P* < 0.0001). A higher OS rate was observed in the nivolumab vs placebo group at 1 (27.3% vs 11.6%) and 2 years (10.6% vs 3.2%). The OS benefit was observed regardless of tumor PD-L1 expression. Among patients with a complete or partial response (CR or PR) in the nivolumab group, the median OS (95% CI) was 26.6 (21.65—not applicable) months; the OS rates at 1 and 2 years were 87.1% and 61.3%, respectively. No new safety signals were identified.

**Conclusions:**

Nivolumab treatment resulted in clinically meaningful long-term improvements in OS in patients with previously treated G/GEJ cancer. The long-term survival benefit of nivolumab was most evident in patients with a CR or PR.

**Electronic supplementary material:**

The online version of this article (10.1007/s10120-019-01034-7) contains supplementary material, which is available to authorized users.

## Introduction

Over 1,000,000 new cases of gastric/gastroesophageal junction (G/GEJ) cancer were reported in 2018 [1], and it is responsible for an estimated 783,000 deaths (equating to one in every 12 deaths) worldwide. It is the fifth most frequently diagnosed cancer and the third leading cause of cancer death globally. The incidences of G/GEJ cancer are markedly higher in eastern Asia, including Japan and Korea [1]. Korea has the highest rates of G/GEJ worldwide in both sexes [1]. The cumulative risk of developing gastric cancer from birth to age 74 is higher in males (1.87%) than in females (0.79%) [1]. Age-standardized rates by sex for G/GEJ cancers in eastern Asia in 2018 were 32.1/100,000 persons in men and 13.2/100,000 persons in women.

Nivolumab, an immune checkpoint inhibitor, was evaluated in the phase 3 ATTRACTION-2 study in patients with G/GEJ cancer treated with ≥ 2 prior chemotherapy regimens [2]. This study previously reported a median overall survival (OS) of 5.26 months with nivolumab vs 4.14 months with placebo. The OS rates at 12 months were 26.2% and 10.9% and the progression-free survival (PFS) rates were 7.6% and 1.5% with nivolumab and placebo, respectively [2]. Consequently, based on the results of ATTRACTION-2 [2], nivolumab is currently approved in Japan [3], South Korea [4], Taiwan [5], Singapore [6], and Switzerland [7] as a third- or later-line therapeutic option in heavily pretreated patients with unresectable advanced or recurrent G/GEJ cancer. Nivolumab is also recommended as third- or later-line therapy in the guidelines for treatment of gastric cancer 2018 in Japan and Korea [8, 9].

Currently, evidence for standard-of-care in third- or later-line therapy for patients with advanced G/GEJ cancer is limited. This includes studies such as KEYNOTE-059 [10], INTEGRATE [11], TAGS [12], JAVELIN Gastric 300 [13], and a Chinese apatinib study [14]. Most of the studies do not provide evidence of long-term efficacy in patients with G/GEJ cancer, with the exception of the phase 2 KEYNOTE-059 study that evaluated the long-term efficacy and safety of pembrolizumab, another immune checkpoint inhibitor [15].

Thus, limited long-term data of immune checkpoint inhibitors exist in advanced G/GEJ cancer, while long-term survival benefits of nivolumab have been reported in other types of malignant diseases [16–22]. Herein, we report the 2-year follow-up results of ATTRACTION-2 (data cutoff, February 18, 2018). Because the durability of the survival benefits, especially in patients achieving objective tumor response remains unclear, we also performed an analysis of OS by best overall response (BOR).

## Methods

### Study design

ATTRACTION-2 is a randomized, double-blind, placebo-controlled, phase 3 study conducted at 49 sites in Japan, South Korea, and Taiwan. The methods have been published previously [2]. In brief, eligible patients were randomized (2:1) to receive nivolumab or placebo. Randomization was stratified according to country (Japan vs Korea vs Taiwan), number of organs with metastases (< 2 vs ≥ 2), and Eastern Cooperative Oncology Group (ECOG) performance status (0 vs 1). The protocol and its amendments were approved by the independent ethics committee or institutional review board at each study center. Written informed consent was provided by all patients before enrollment, and a separate written consent was obtained for collection of tumor tissue for biomarker analysis. The study was conducted in accordance with the Declaration of Helsinki and the Good Clinical Practice guidelines developed by the International Council for Harmonisation of Technical Requirements for Pharmaceuticals for Human Use.

### Patients

Patients aged 20 years or older with unresectable advanced or recurrent G/GEJ cancer, histologically confirmed to be adenocarcinoma refractory to or intolerant of standard therapy, were eligible for inclusion in the study. Patients must have received treatment with two or more lines of previous chemotherapy in the advanced or recurrent setting, have an ECOG performance status of 0 or 1, and a life expectancy of at least 3 months. Patients previously treated with anti-programmed death-1 (PD-1), anti-programmed death ligand-1 (PD-L1) or anti-PD-L2, anti-CD137, or anti-cytotoxic T-lymphocyte-associated protein-4 (CTLA-4) antibodies were excluded. Further details of exclusion criteria are mentioned in the previous publication [2].

### Treatment and assessments

Patients received an intravenous infusion of nivolumab (3 mg/kg) or placebo every 2 weeks for 6 weeks (one treatment cycle). Study treatment was continued until disease progression or the onset of toxicities requiring permanent treatment discontinuation. After initial evidence of disease progression, patients could continue the study treatment provided the following criteria were met: evidence of clinical benefit, tolerance for the study drug and stable performance status, treatment continuation not impacting any interventions required to prevent serious complications by disease progression, and provision of written informed consent to continue study treatment by the patient. The minimum follow-up period was defined as the time from randomization of the last patient to data cutoff.

The primary endpoint was OS. Secondary efficacy endpoints were PFS, objective response rate [ORR; proportion of patients with confirmed complete response (CR) or partial response (PR)], disease control rate [proportion of patients with confirmed CR, PR, or stable disease (SD)], and BOR [CR + PR, SD, and progressive disease (PD)]. Tumor responses were assessed with computed tomography (CT) or magnetic resonance imaging (MRI) after each treatment cycle for first ten cycles and after every two treatment cycles thereafter until discontinuation of study treatment or the initiation of the poststudy treatment. Tumors were assessed according to the Response Evaluation Criteria in Solid Tumors (RECIST) guidelines version 1.1 [23].

Adverse events (AEs) were evaluated using the National Cancer Institute Common Terminology Criteria for Adverse Events version 4.0 [24] during treatment (+ 28 days). Incidences of treatment-related adverse events (TRAEs) of special interest (AEs of special clinical interest with a potential immune-related etiology) were also evaluated.

Tumor tissue collection was not mandatory, and exploratory analysis of PD-L1 expression (PD-L1 positivity: 1% or more of tumor cells) was performed by a central laboratory using immunohistochemistry (28-8 pharmDx assay; Dako, Carpinteria, CA, USA) on the available tumor samples. Exploratory subanalysis of OS was performed in patients with CR + PR, SD, and PD.

Additionally, an exploratory landmark analysis was performed in patients who had SD at the first 6-week assessment. Patients with SD at the first 6-week assessment were categorized into the following three groups based on the tumor growth rate at 6 weeks: group 1 (− 30% < and ≤  − 5%), group 2 (− 5% < and <  + 5%), and group 3 (+ 5% ≤ and <  + 20%); OS curves were generated from 6 weeks onwards. The tumor growth rate was calculated as a change in tumor volume from baseline in the 6-week period. Since no definitive cutoff value was specified for this categorization, this landmark analysis was exploratory.

### Statistical analysis

Sample size estimation has been described previously [2]. OS and PFS were compared between the treatment groups using the stratified log-rank test with a one-sided significance level of 0.025. Hazard ratio [HR; 95% confidence interval (CI)] was calculated using the stratified Cox proportional hazards model. The Kaplan–Meier method was used to estimate the median OS and median PFS, and for the subanalysis of OS by BOR and by tumor PD-L1 expression status. For the landmark analysis, standard OS curves were generated for patients found to have SD at the first evaluation (6 weeks), and the patients were categorized into three groups based on the tumor growth rate at 6 weeks. All analyses were performed using SAS versions 9.3 and 9.4 (SAS Institute, Inc., Cary, NC, USA).

## Results

### Patient disposition and baseline characteristics

Overall, 601 patients were screened, of whom 493 (nivolumab, 330; placebo, 163) were randomized in ATTRACTION-2. The safety assessment population comprised 491 patients (nivolumab, 330; placebo, 161), and the response assessment population comprised 399 patients with measurable lesions (nivolumab, 268; placebo, 131; data cutoff, February 18, 2018). Further details of patient disposition have been reported previously [2]. The median age (interquartile range [IQR]) and proportion of men were 62 (54–69) years and 69.4% in the nivolumab group and 61 (53–68) years and 73% in the placebo group, respectively. No substantial difference was observed in the baseline characteristics between the nivolumab and placebo groups (Online Resource Table 1).

### Exposure and subsequent pharmacotherapy

The median (min–max) duration of treatment was 1.92 (0.0–28.4) months with nivolumab and 1.05 (0.0–29.9) months with placebo. Overall, the relative dose intensity of nivolumab was 90% to < 110% in 79.4% of patients. Details of the study drug exposure and administration are presented in Online Resource Table 2.

At data cutoff, study treatment was permanently discontinued in 322 patients (97.6%) in the nivolumab group and in 161 patients (98.8%) in the placebo group. Reasons for treatment discontinuation (nivolumab vs placebo, respectively) were as follows: disease progression (237 [71.8%] vs 109 [67.7%]), worsening of clinical symptoms judged as PD (59 [17.9%] vs 39 [24.2%]), onset of grade ≥ 2 interstitial lung disease (5 [1.5%] vs 0 [0%]), physician discretion (13 [3.9%] vs 3 [1.9%]), treatment withheld longer than 6 weeks due to AEs (7 [2.1%] vs 1 [0.6%]), and other reasons (27 [8.2%] vs 19 [11.8%]).

Following study treatment discontinuation, 53.6% (177/330) and 47.2% (77/163) of patients in the nivolumab and placebo groups, respectively, received subsequent anticancer treatment (pharmacotherapy, 41.5% [137/330] and 35% [57/163]; surgery, 20.9% [69/330] and 17.2% [28/163]; radiotherapy, 8.5% [28/330] and 10.4% [17/163]; Online Resource Table 3). Among all patients, 109 (33%) patients in the nivolumab group and 37 (23%) patients in the placebo group continuously received the study treatment after being judged as having PD as per RECIST version 1.1. In total, six (1.8%) patients in the nivolumab group and two (1.2%) patients in the placebo group received immune checkpoint inhibitors as subsequent therapy.

### Efficacy

**Fig. 1:**
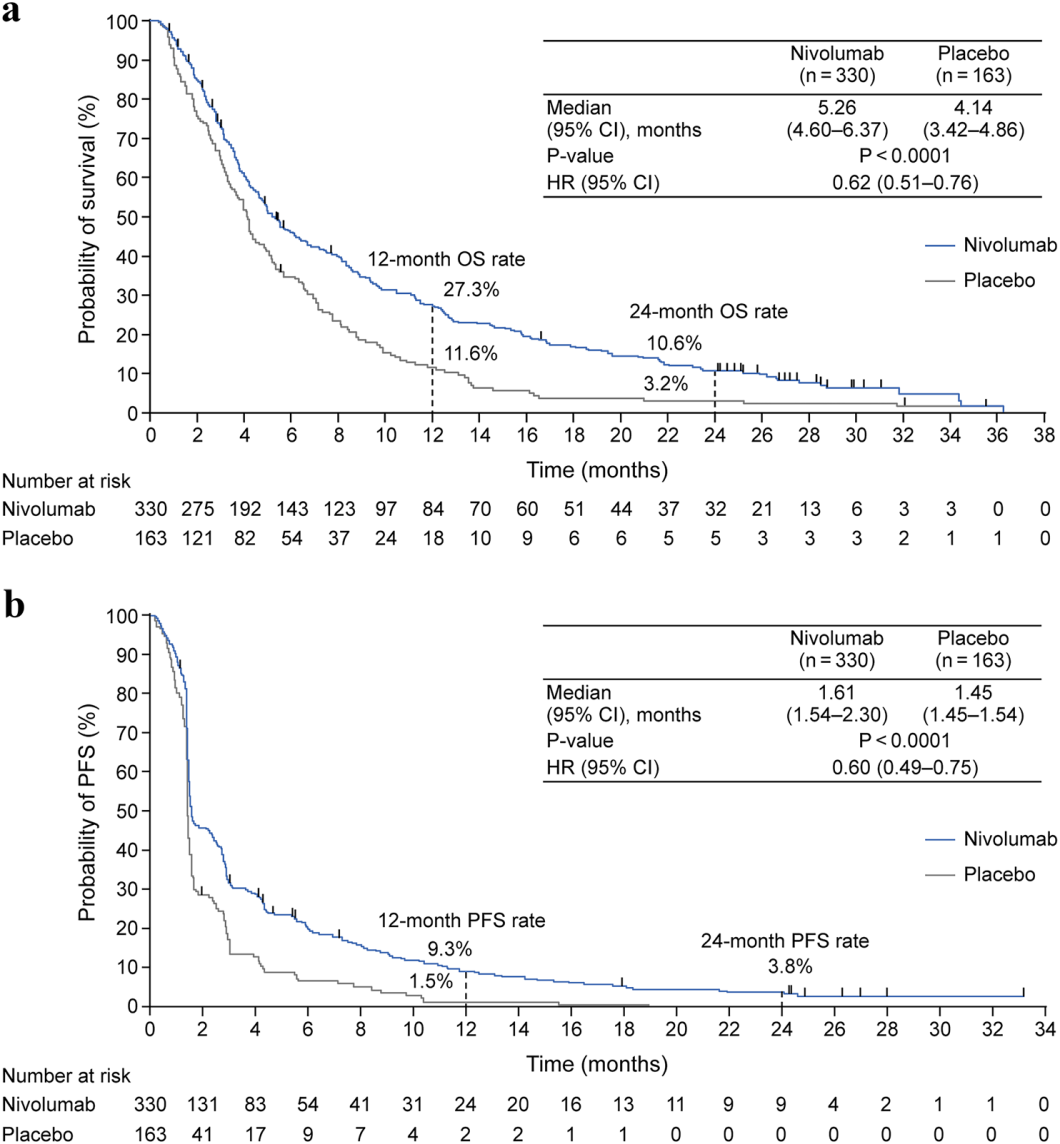
Kaplan–Meier plot of OS (**a**) and PFS (**b**) after 2 years of follow-up. Marks on the curve indicate patients who were censored. *CI* confidence interval, *HR* hazard ratio, *OS* overall survival, *PFS* progression-free survival

The median OS (95% CI) in the nivolumab vs placebo group was 5.26 (4.60–6.37) vs 4.14 (3.42–4.86) months at the 2-year follow-up. The OS rate was longer in the nivolumab group than in the placebo group throughout the study period [2]. The risk of death was significantly lower in the nivolumab group than in the placebo group (HR [95% CI], 0.62 [0.51–0.76]; *P* < 0.0001; Fig. 1a). A higher OS rate was also observed in the nivolumab group compared with the placebo group at 1 year (27.3% vs 11.6%) and 2 years (10.6% vs 3.2%).

The median PFS (95% CI) in the nivolumab group compared with the placebo group was 1.61 (1.54–2.30) vs 1.45 (1.45–1.54) months at the 2-year follow-up. The PFS rate was higher in the nivolumab group than in the placebo group after approximately 2 months of treatment initiation throughout the study period [2]. The risk of disease progression was lower in the nivolumab than in the placebo group (HR [95% CI], 0.60 [0.49–0.75]; *P* < 0.0001; Fig. 1b). The PFS rate at 1 year was higher in the nivolumab group compared with the placebo group (9.3% vs 1.5%); at 2 years, the PFS rate was 3.8% in the nivolumab group, and disease progression was reported in all patients in the placebo group. Subgroup analyses of OS according to baseline demographics and disease characteristics consistently favored nivolumab over placebo (Online Resource Fig. 1).

### BOR

The ORR was greater in the nivolumab group than in the placebo group, with a CR or PR observed in 32 patients (11.9%; CR, 3 [1.1%] and PR, 29 [10.8%]) compared with no patients, respectively, at the 2-year follow-up (median [IQR], 27.2 [25.2–29.9] months). Of note, no CRs and 30 PRs had been observed at the initial follow-up (median, 8.9 months; ORR: nivolumab, 11.2%; placebo, 0%) [2]. Taken together, three cases of PR at the 1-year follow-up transitioned to CR at the 2-year follow-up. BOR is described in Table 1. All three patients in the nivolumab group were evaluated as CR at assessment of week 66 with 11 cycles of nivolumab after they had shown PR at the 1-year follow-up. All three patients with CR at the 2-year follow-up had a baseline ECOG performance status of 1 with liver or lung metastasis and extensive lymph nodal, including supraclavicular node, metastasis. The details of the three patients with CR are shown in Online Resource Table 4.

**Table 1:** Best overall response in the overall population

*n* (%)	Overall population	
	Nivolumab (*n* = 268)	Placebo (*n* = 131)
Best overall response		
CR	3 (1.1)	0
PR	29 (10.8)	0
SD	76 (28.4)	33 (25.2)
PD	124 (46.3)	79 (60.3)
NE	36 (13.4)	19 (14.5)
Objective response rate (CR or PR)	32 (11.9)	0
Disease control rate (CR, PR, or SD)	108 (40.3)	33 (25.2)

*CR* complete response, *NE* not evaluable, *PD* progressive disease, *PR* partial response, *SD* stable disease

### Subanalysis of OS by BOR

**Fig. 2:**
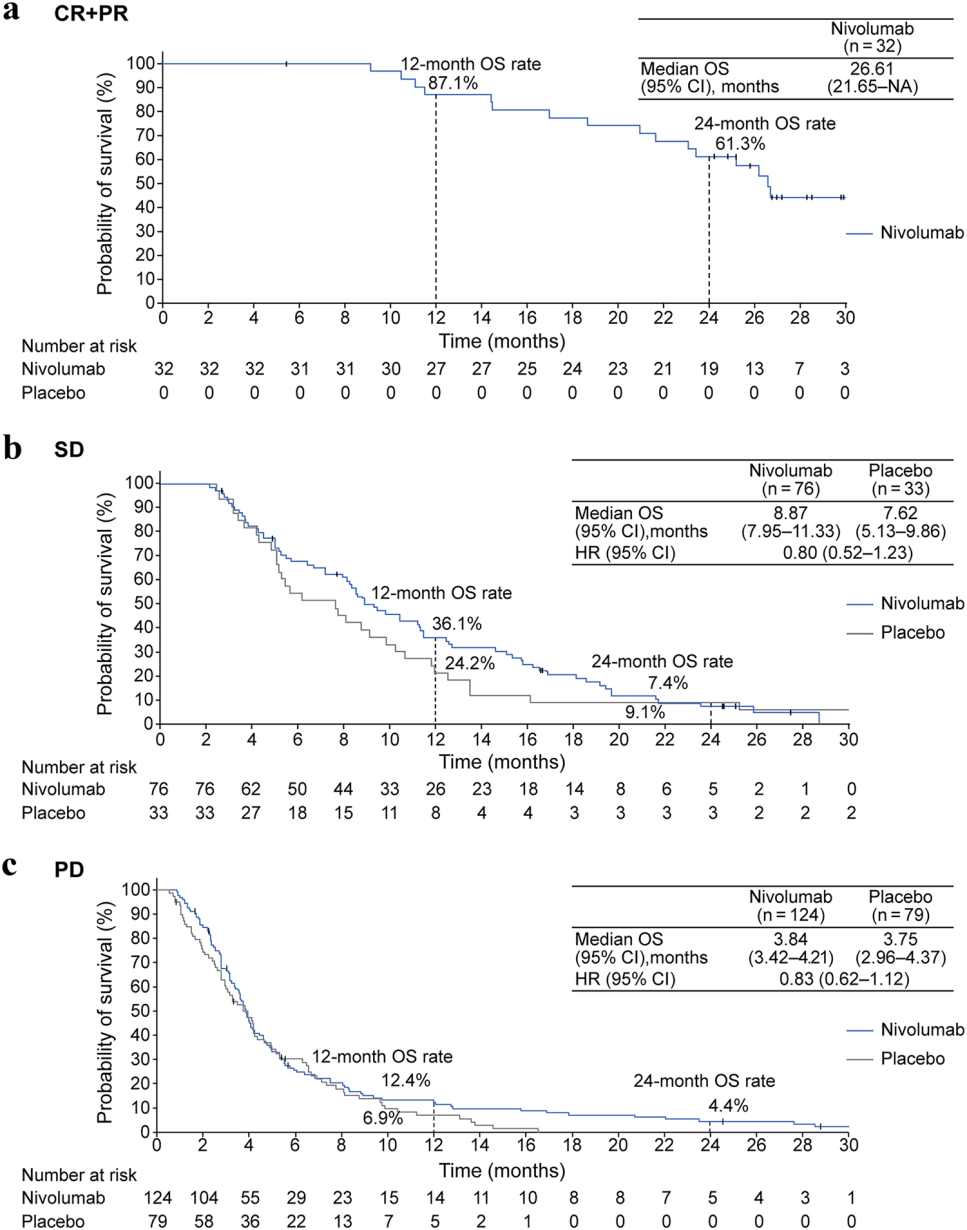
Subanalysis of OS by BOR among patients with CR + PR (**a**), SD (**b**), and PD (**c**). Marks on the curve indicate patients who were censored. *BOR* best overall response, *CI* confidence interval, *CR* complete response, *HR* hazard ratio, *NA* not applicable, *OS* overall survival, *PD* progressive disease, *PR* partial response, *SD* stable disease

Among patients with a CR or PR in the nivolumab group, the median (95% CI) OS was 26.6 (21.65—not applicable) months; the OS rates at 1 and 2 years were 87.1% and 61.3%, respectively. No patient in the placebo group had a CR or PR (Fig. 2a). Results of the subanalysis by BOR showed that among patients with SD, a marginally longer OS was also observed (median [95% CI]: nivolumab, 8.87 [7.95–11.33] months; placebo, 7.62 [5.13–9.86] months; HR [95% CI], 0.80 [0.52–1.23]; Fig. 2b). The survival curves for patients with PD overlapped within 1 year, while five patients in the nivolumab group survived longer than 2 years (median [95% CI] OS: nivolumab, 3.84 [3.42–4.21] months; placebo, 3.75 [2.96–4.37] months; HR [95% CI], 0.83 [0.62–1.12]; Fig. 2c). All of these five patients received post-progression anticancer therapies, and three of them continued nivolumab after disease progression. One patient showed some tumor shrinkage beyond PD.

### Exploratory analysis

Exploratory analysis based on PD-L1 expression status showed that median (95% CI) OS in patients with PD-L1-positive tumors was 5.22 (2.79–9.36) months in the nivolumab group and 3.83 (0.79–4.96) months in the placebo group (HR [95% CI], 0.75 [0.32–1.72]; Online Resource Fig. 2a). In patients with PD-L1-negative tumors, median (95% CI) OS was 6.05 (4.83–8.61) months in the nivolumab group and 4.19 (3.02–6.93) months in the placebo group (HR [95% CI], 0.70 [0.50–0.99]; Online Resource Fig. 2b). The OS benefit was observed regardless of PD-L1 expression status as reported previously [2].

Among patients in whom response could be evaluated, the exploratory landmark analysis showed that in patients with SD at the first 6-week assessment, difference in the median OS between the nivolumab and placebo groups was 8.81, 3.55, and 3.15 months in the tumor growth rate group 1 (− 30% < and ≤  − 5%), group 2 (− 5% < and <  + 5%), and group 3 (+ 5% ≤ and <  + 20%), respectively. The OS rate and curves in the nivolumab group were slightly better than those in the placebo group across the three tumor growth rate groups (Online Resource Fig. 3).

### Safety

**Fig. 3:**
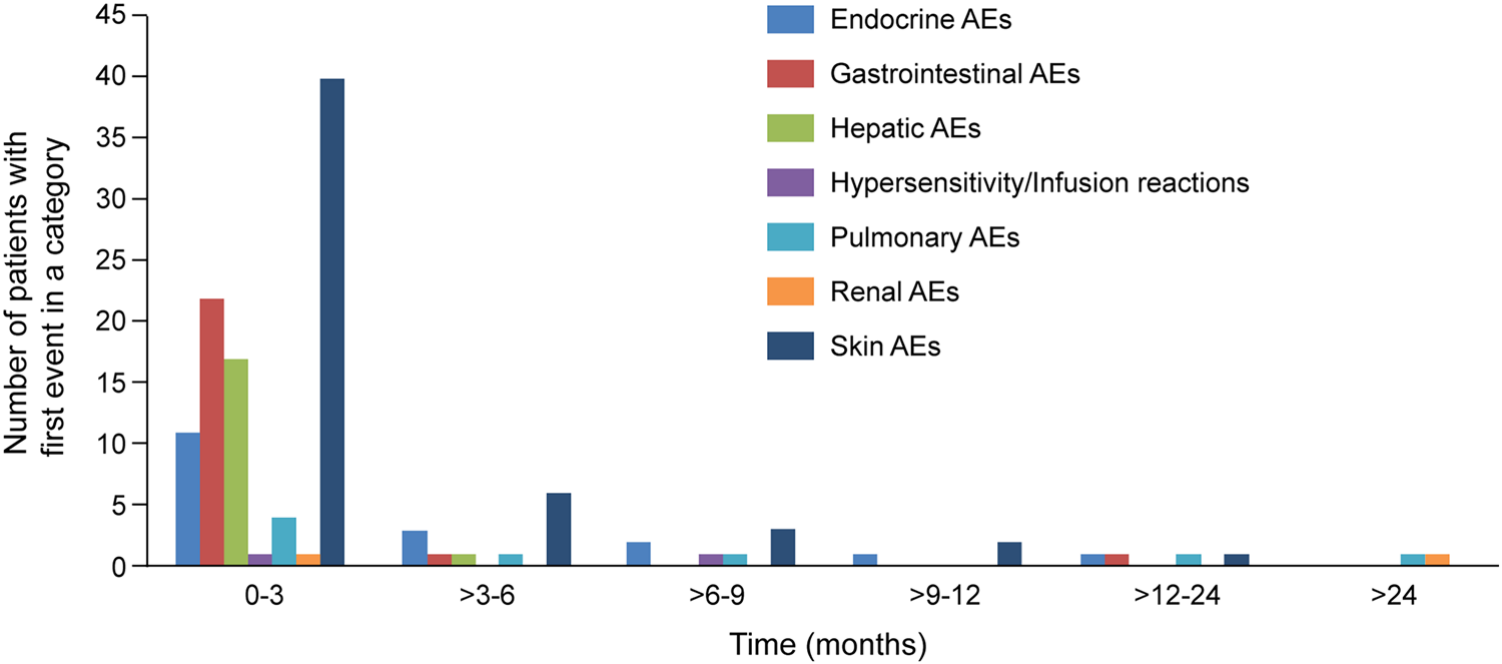
Emergence of treatment-related AEs (any grade) of special interest over time. *AE* adverse event

Safety analyses were performed in 330 patients in the nivolumab group and 161 patients in the placebo group who received one or more doses of nivolumab. All-cause AEs of any grade were reported in 301 (91.2%) of 330 patients in the nivolumab group and 135 (83.9%) of 161 patients in the placebo group. TRAEs of any grade were reported in 142 (43.0%) patients in the nivolumab group and 43 (26.7%) patients in the placebo group, including 39 (11.8%) and seven (4.3%) patients with grade 3–4 TRAEs, respectively. Serious TRAEs were reported in 38 (11.5%) of 330 patients in the nivolumab group and eight (5.0%) of 161 patients in the placebo group. Most patients experienced onset of TRAEs of special interest within 3 months of starting nivolumab: skin, gastrointestinal, hepatic, and endocrine TRAEs were most commonly experienced at 3 months and tended to abate over time. The incidence rates of TRAEs of special interest were comparable at 6 months, 1 year, and 2 years. No major late-onset TRAEs were observed (Fig. 3). Among TRAEs of special interest, one additional case each of maculopapular rash and pneumonitis was observed during the additional follow-up period, compared with the previous publication [2] (Online Resource Table 5).

## Discussion

Large-scale clinical trials of third-line treatment for advanced/recurrent G/GEJ cancer are limited. The results of this long-term follow-up of ATTRACTION-2 [2] demonstrated that compared with placebo, nivolumab significantly prolonged the OS (5.26 vs 4.14 months), with numerically higher OS (10.6% vs 3.2%) and PFS rates (3.8% vs 0%) at 2 years in patients with unresectable advanced or recurrent G/GEJ cancer after two or more prior chemotherapy regimens. While the OS rates were higher in the nivolumab group than in the placebo group throughout the study period, higher PFS rates favoring nivolumab became evident after approximately 2 months of treatment initiation [2].

Furthermore, treatment discontinuation rate due to AEs was low, and no new safety signals were identified compared with previous reports in patients with various cancer types [2, 16, 19, 21, 25–28]. Most patients experienced their first onset of TRAE of special interest (immune-related) within 3 months of starting nivolumab. Thereafter, the incidence of TRAEs of special interest was low and tended to abate over time, suggesting a favorable long-term tolerability profile for continued nivolumab therapy. However, monitoring is recommended to identify any potential late-onset AEs.

In the nivolumab group, there were 32 patients with CR or PR. In the OS subanalysis by BOR, a median OS of 26.6 months was observed in these patients. The number of patients with CR increased from zero to three during the 2-year follow-up. The three patients with CR did not demonstrate any specific background characteristics.

The results showed that the survival benefit over the 2-year follow-up period was observed regardless of PD-L1 expression status, as reported previously [2]. Limitations of this study are that the exploratory analysis of tumor PD-L1 expression status was performed in a limited number of patients, and PD-L1 expression was analyzed only in tumor cells.

A total of 76 patients (28.4%) on nivolumab had SD. Patients with SD at 6 weeks had a range of tumor growth rate from − 30 to + 20%, meaning either a slight decrease or a slight increase. We performed an exploratory analysis that assessed OS among SD patients by subgrouping them based on the tumor growth rate at the first assessment (6 weeks; RECIST criteria) to examine whether or not the continued use of nivolumab could provide clinical benefit to SD patients even after a slight increase in tumor size. When categorized by three tumor growth rate groups (− 30% < and ≤  − 5%; − 5% < and <  + 5%; + 5% ≤ and <  + 20%) in these patients with SD at 6 weeks, the OS rate/curves in the nivolumab group were higher than those in the placebo group across the three tumor growth rate groups. Comparing OS in the subset showing SD at 6 weeks might provide further insights into the efficacy of nivolumab. Patients with SD in the placebo group had more indolent and slow-growing tumors compared with patients with PD, but some of the patients with SD in the nivolumab group might have had aggressive tumors whose growth could be inhibited by nivolumab. Furthermore, in the phase III trial of nivolumab in non-small cell lung cancer, discontinuation of nivolumab after disease control for 1 year resulted in poor prognosis compared with its continuation [29]. It is suggested that continuous therapy with nivolumab could still be a viable treatment option even after a small increase in tumor size within SD.

All five of the patients with PD at initial response assessment who survived longer than 2 years received post-progression therapy, and three of them continued nivolumab beyond PD. Only one patient showed tumor shrinkage with nivolumab beyond PD. While this study allowed continuation of nivolumab beyond PD conditionally, the clinical significance of this treatment is not clear.

Overall, the results of OS by BOR should be interpreted with caution because clinical significance of continuing nivolumab should be confirmed in a randomized trial. Furthermore, other prognostic factors, including natural tumor growth kinetics (i.e., slow tumor progression with good prognosis), may have influenced the outcome in patients with SD.

## Conclusions

The efficacy of nivolumab was similar to and sustained from the 1-year follow-up as demonstrated by continued clinically meaningful improvements in OS and PFS at the 2-year follow-up compared with placebo. The long-term survival benefit of nivolumab was most evident in patients with CR or PR than in those with SD or PD. Even among patients with SD categorized by tumor growth rate, nivolumab offered a longer median OS than placebo, suggesting that nivolumab can be continued even after a small increase in tumor size within SD. However, these observations will need to be validated in future studies by evaluating the use of nivolumab in beyond-PD cases. The safety profile was similar to that at the 1-year follow-up, and no major late-onset TRAEs of special interest were observed; however, continual monitoring of AEs was necessary.

## Electronic supplementary material

Below is the link to the electronic supplementary material.

Supplementary file1 (DOCX 1539 kb)
