# Supplementary material for: A phase 3 study of nivolumab in previously treated advanced gastric or gastroesophageal junction cancer (ATTRACTION-2): 2-year update data
Source: Gastric Cancer. 2019 Dec 20;23(3):510–9. doi: 10.1007/s10120-019-01034-7 (PMC7165140; doi:10.1007/s10120-019-01034-7)

**Title:** A Phase 3 Study of Nivolumab in Previously Treated Advanced Gastric or Gastroesophageal Junction Cancer (ATTRACTION-2): 2-Year Update Data

**Journal:** Gastric cancer

**Authors:**

Li-Tzong Chen^1^, Taroh Satoh^2^, Min-Hee Ryu^3^, Yee Chao^4^, Ken Kato^5^, Hyun Cheol Chung^6^, Jen-Shi Chen^7^, Kei Muro^8^, Won Ki Kang^9^, Kun-Huei Yeh^10^, Takaki Yoshikawa^11^, Sang Cheul Oh^12^, Li-Yuan Bai^13^, Takao Tamura^14^, Keun-Wook Lee^15^, Yasuo Hamamoto^16^, Jong Gwang Kim^17^, Keisho Chin^18^, Do-Youn Oh^19^, Keiko Minashi^20^, Jae Yong Cho^21^, Masahiro Tsuda^22^, Hiroki Sameshima^23^, Yoon-Koo Kang^3^, Narikazu Boku^5^

**Affiliations:**

^1^National Institute of Cancer Research, National Health Research Institutes, and National Cheng Kung University Hospital, National Cheng Kung University, Tainan, Taiwan

^2^Frontier Science for Cancer and Chemotherapy, Osaka University Graduate School of Medicine, Suita, Japan

^3^Department of Oncology, University of Ulsan College of Medicine, Asan Medical Center, Seoul, South Korea

^4^Department of Oncology, Taipei Veterans General Hospital, Taipei, Taiwan

^5^Division of Gastrointestinal Medical Oncology, National Cancer Center Hospital, Tokyo, Japan

^6^Division of Medical Oncology, Yonsei Cancer Center, Song-Dang Institute for Cancer Research, Yonsei University College of Medicine, Yonsei University Health System, Seoul, South Korea

^7^Division of Hematology and Oncology, Department of Internal Medicine, Linkou Chang Gung Memorial Hospital, Chang Gung University, Taoyuan, Taiwan

^8^Department of Clinical Oncology, Aichi Cancer Center Hospital, Nagoya, Japan

^9^Division of Hematology-Oncology, Department of Medicine, Samsung Medical Center, Sungkyunkwan University School of Medicine, Seoul, South Korea

^10^National Taiwan University Cancer Center; and Department of Oncology, National Taiwan University Hospital, National Taiwan University College of Medicine, Taipei, Taiwan

^11^Department of Gastrointestinal Surgery, Kanagawa Cancer Center, Yokohama, Japan

^12^Division of Hematology and Oncology, Department of Internal Medicine, College of Medicine, Korea University, Seoul, South Korea

^13^Division of Hematology and Oncology, Department of Internal Medicine, China Medical University Hospital, China Medical University, Taichung, Taiwan

^14^Department of Medical Oncology, Faculty of Medicine, Kindai University, Osakasayama, Japan

^15^Division of Hematology and Oncology, Department of Internal Medicine, Seoul National University Bundang Hospital, Seoul National University College of Medicine, Seongnam, South Korea

^16^Keio Cancer Center, Keio University School of Medicine, Tokyo, Japan

^17^Kyungpook National University School of Medicine, Daegu, South Korea

^18^Department of Gastroenterology, Cancer Institute Hospital of the Japanese Foundation for Cancer Research, Tokyo, Japan

^19^Department of Internal Medicine, Seoul National University Hospital, Seoul, South Korea

^20^Clinical Trial Promotion Department, Chiba Cancer Center, Chiba, Japan

^21^Department of Medical Oncology, Gangnam Severance Hospital, Yonsei University College of Medicine, Seoul, South Korea

^22^Department of Gastroenterological Oncology, Hyogo Cancer Center, Akashi, Japan

^23^Medical Oncology, Medical Affairs, Ono Pharmaceutical Co., Ltd., Osaka, Japan

**Corresponding author:** Narikazu Boku

Email: [nboku@ncc.go.jp](mailto:nboku@ncc.go.jp)

**Supplementary data**

**Online Resource Table 1** Demographics and baseline characteristics

|  | **Nivolumab (3 mg/kg)**  **(n=330)** | **Placebo**  **(n=163)** |
| --- | --- | --- |
| Median age (IQR), years | 62 (54–69) | 61 (53–68) |
| <65 years, n (%) | 189 (57.3) | 95 (58.3) |
| Male, n (%) | 229 (69.4) | 119 (73.0) |
| Country, n (%) |  |  |
| Japan | 152 (46.1) | 74 (45.4) |
| Korea | 146 (44.2) | 74 (45.4) |
| Taiwan | 32 (9.7) | 15 (9.2) |
| ECOG PS, n (%) |  |  |
| 0 | 95 (28.8) | 48 (29.4) |
| 1 | 235 (71.2) | 115 (70.6) |
| Primary site of disease, n (%) |  |  |
| Gastric | 272 (82.4) | 135 (82.8) |
| Gastroesophageal junction | 30 (9.1) | 12 (7.4) |
| Unknown | 28 (8.5) | 16 (9.8) |
| Prior gastrectomy, n (%) |  |  |
| No | 133 (40.3) | 58 (35.6) |
| Yes | 197 (59.7) | 105 (64.4) |
| Organs with metastases (≥2), n (%) | 246 (74.5) | 119 (73.0) |
| Prior treatment regimens, n (%) |  |  |
| 2 | 69 (20.9) | 29 (17.8) |
| 3 | 137 (41.5) | 62 (38.0) |
| ≥4 | 124 (37.6) | 72 (44.2) |
| Any prior therapy, n (%) | 330 (100.0) | 163 (100.0) |
| Fluoropyrimidine | 329 (99.7) | 163 (100.0) |
| Platinum | 311 (94.2) | 157 (96.3) |
| Taxane | 284 (86.1) | 140 (85.9) |
| Irinotecan | 247 (74.8) | 123 (75.5) |
| Ramucirumab | 35 (10.6) | 22 (13.5) |

*ECOG PS* Eastern Cooperative Oncology Group performance status, *IQR* interquartile range.

**Online Resource Table 2** Extent of exposure and administration of study treatment (SAF)

|  | **Nivolumab** | **Placebo** |
| --- | --- | --- |
| Analysis item (unit) | n (%) | n (%) |
|  | N=330 | N=161 |
| Number of doses received (times) |  |  |
| Mean (StD) | 9.3 (12.4) | 5.2 (7.1) |
| Median | 5.0 | 3.0 |
| Min–max | 1–60 | 1–61 |
| Duration of treatment (months)^a^ |  |  |
| >6 | 65 (19.7) | 9 (5.6) |
| >12 | 33 (10.0) | 3 (1.9) |
| Mean (StD) | 4.17 (6.08) | 2.12 (3.47) |
| Median | 1.92 | 1.05 |
| Min–max | 0.0–28.4 | 0.0–29.9 |
| Number of cycles^b^ |  |  |
| 1 | 144 (43.6) | 89 (55.3) |
| 2–3 | 103 (31.2) | 60 (37.3) |
| 4–6 | 39 (11.8) | 6 (3.7) |
| ≥7 | 44 (13.3) | 6 (3.7) |
| Mean (StD) | 3.4 (4.2) | 2.0 (2.4) |
| Median | 2.0 | 1.0 |
| Min–max | 1–22 | 1–21 |
| Cumulative dose, mg/kg^c^ |  |  |
| Mean (StD) | 27.92 (37.23) |  |
| Median | 14.49 |  |
| Min–max | 3.0–185.8 |  |
| Relative dose intensity, %^d^ |  |  |
| <50 | 1 (0.3) |  |
| 50–<70 | 6 (1.8) |  |
| 70–<90 | 59 (17.9) |  |
| 90–<110 | 262 (79.4) |  |
| ≥110 | 2 (0.6) |  |
| Mean (StD) | 95.09 (8.29) |  |
| Median | 96.85 |  |
| Min–max | 46.7–112.6 |  |

^a^Duration of treatment (months) = (“Date of the last dose” – “Date of the first dose” + 1)/30.4375

^b^The number of cycles will be calculated for the cycle proceeding to the next cycle. The discontinued cycle or the cycle receiving no investigational product also will be included

^c^Cumulative dose of nivolumab (mg/kg) is sum of the actual doses (mg/kg) administrated to a subject during the treatment period. Actual dose (mg/kg) at each time point will be calculated by the following equation: Actual dose (mg/kg) at each time point = “Actual dose amount (mg)”/“Recent weight (kg)”

^d^Relative dose intensity (%) = “Cumulative dose (mg/kg)”/“[Date of the last dose – Date of the first dose + 14] (days) × 3 (mg/kg)/14 (days)” × 100

*SAF* safety analysis set, *StD* standard deviation

**Online Resource Table 3** Postprogression anticancer therapies (ITT population)

| **Therapy, n (%)** | **Nivolumab** | **Placebo** |
| --- | --- | --- |
|  | **(N=330)** | **(N=163)** |
| Any postprogression therapy | 177 (53.6) | 77 (47.2) |
| Radiotherapy | 28 (8.5) | 17 (10.4) |
| Surgery | 69 (20.9) | 28 (17.2) |
| Pharmacotherapy | 137 (41.5) | 57 (35.0) |
| Postprogression pharmacotherapy |  |  |
| Fluoropyrimidine | 44 (13.3) | 25 (15.3) |
| Platinum compound | 30 (9.1) | 18 (11.0) |
| Taxane | 35 (10.6) | 16 (9.8) |
| Irinotecan | 18 (5.5) | 9 (5.5) |
| Ramucirumab | 45 (13.6) | 12 (7.4) |
| Immunotherapy^a^ | 6 (1.8) | 3 (1.8) |
| Other targeted therapies^b^ | 8 (2.4) | 5 (3.1) |

^a^Includes atezolizumab, MOXR0916, nivolumab, pembrolizumab, and immunotherapy (not specified)

^b^Afatinib, bevacizumab, AZD6738, cetuximab, FPA144, GC1118, GDC-0068, gefitinib, JNJ42756493, lapatinib, and trastuzumab

*ITT* intention-to-treat

**Online Resource Table 4** Baseline characteristics of three patients with CR

|  | **Case 1** | **Case 2** | **Case 3** |
| --- | --- | --- | --- |
| Sex | Male | Male | Male |
| Age, years | 83 | 63 | 58 |
| BMI, kg/m^2^ | 22.4 | 24.4 | 27.7 |
| ECOG PS | 1 | 1 | 1 |
| Number of prior regimens | 5 | 3 | 2 |
| Diameters of target lesion, mm | 159.1 | 36 | 178 |
| Target region | Supraclavicular lymph node, liver (left and right lobe) | Para-aortic lymph node | Stomach, liver, pancreas, and para-aortic lymph node |
| Nontarget region | Lung, liver, abdominal cavity, and para-aortic lymph node | Abdominal lymph node,  supraclavicular lymph node, and lung | Para-aortic lymph node and supraclavicular lymph node |
| Microsatellite instability | Negative | Sample not available | Negative |
| PD-L1 status | Negative | Sample not available | Negative |

*BMI* body mass index, *CR* complete response, *ECOG PS* Eastern Cooperative Oncology Group performance status, *PD-L1* programmed death ligand-1

**Online Resource Table 5** Treatment-related adverse events of special interest

| Additional treatment-related adverse events of special interest | | | | |
| --- | --- | --- | --- | --- |
|  | **Nivolumab (n=330)** | | **Placebo (n=161)** | |
|  | Any grade | Grade 3 or 4 | Any grade | Grade 3 or 4 |
| Interstitial lung disease | 6 (1.8) | 1 (0.3) | 0 | 0 |
| Maculopapular rash | 5 (1.5) | 0 | 1 (0.6) | 0 |
| Colitis | 2 (0.6) | 1 (0.3) | 0 | 0 |
| Hyperthyroidism | 2 (0.6) | 0 | 0 | 0 |
| Acute hepatitis | 1 (0.3) | 1 (0.3) | 0 | 0 |
| Autoimmune thyroiditis | 1 (0.3) | 0 | 0 | 0 |
| Hypopituitarism | 1 (0.3) | 1 (0.3) | 0 | 0 |
| Pneumonitis | 2 (0.6) | 1 (0.3) | 0 | 0 |
| Thyroid disorder | 1 (0.3) | 0 | 0 | 0 |

Data are number of patients (%)

**Online Resource Fig. 1** Forest plot of overall survival according to patient subgroups.

HRs for overall survival according to patient subgroups were calculated using the unstratified Cox proportional hazards model. *CI* confidence interval, *ECOG* Eastern Cooperative Oncology Group, *HR* hazard ratio. n=events. N=group size. *Patients with both gastric and esophagogastric junction lesion sites are included in the gastric category. ^†^Includes fundus, corpus, antrum, and pylorus. ^‡^Includes treatments received in the adjuvant setting

**Online Resource Fig. 2** Kaplan-Meier plots of OS based on PD-L1 expression of ≥1% (**a**) and PD-L1 expression of <1% (**b**). Marks on the curve indicate patients who were censored.

*CI* confidence interval, *HR* hazard ratio, *OS* overall survival, *PD-L1* programmed death ligand-1

**Online Resource Fig. 3** Kaplan-Meier plots of OS among patients with SD and tumor growth rate between −30%< and ≤−5%, group 1 (**a**), −5%< and <+5%, group 2 (**b**), and +5%≤ and <+20%, group 3 (**c**). Marks on the curve indicate patients who were censored.

*CI* confidence interval, *OS* overall survival, *SD* stable disease

**Online Resource Fig. 1**


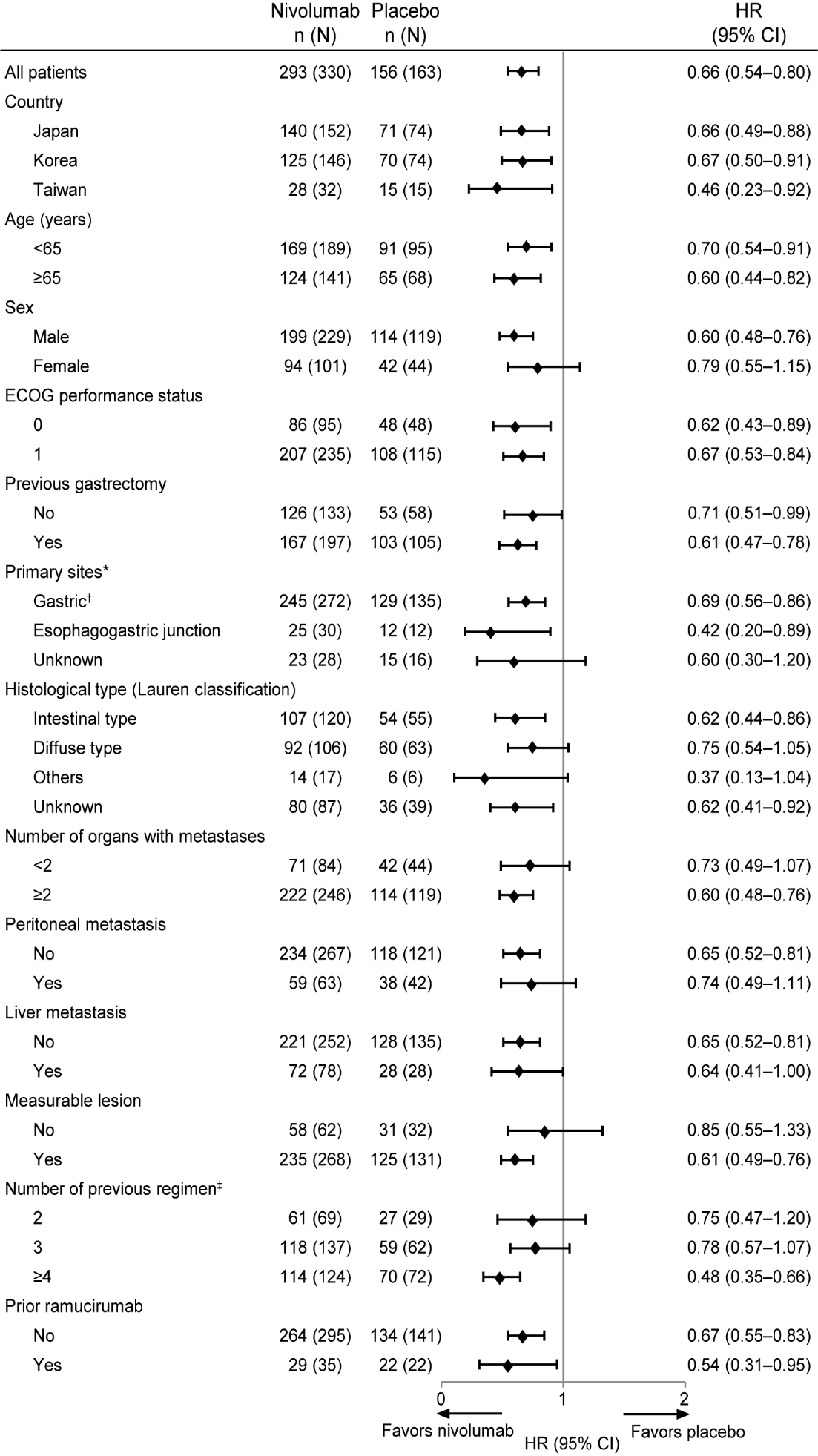


**Online Resource Fig. 2**


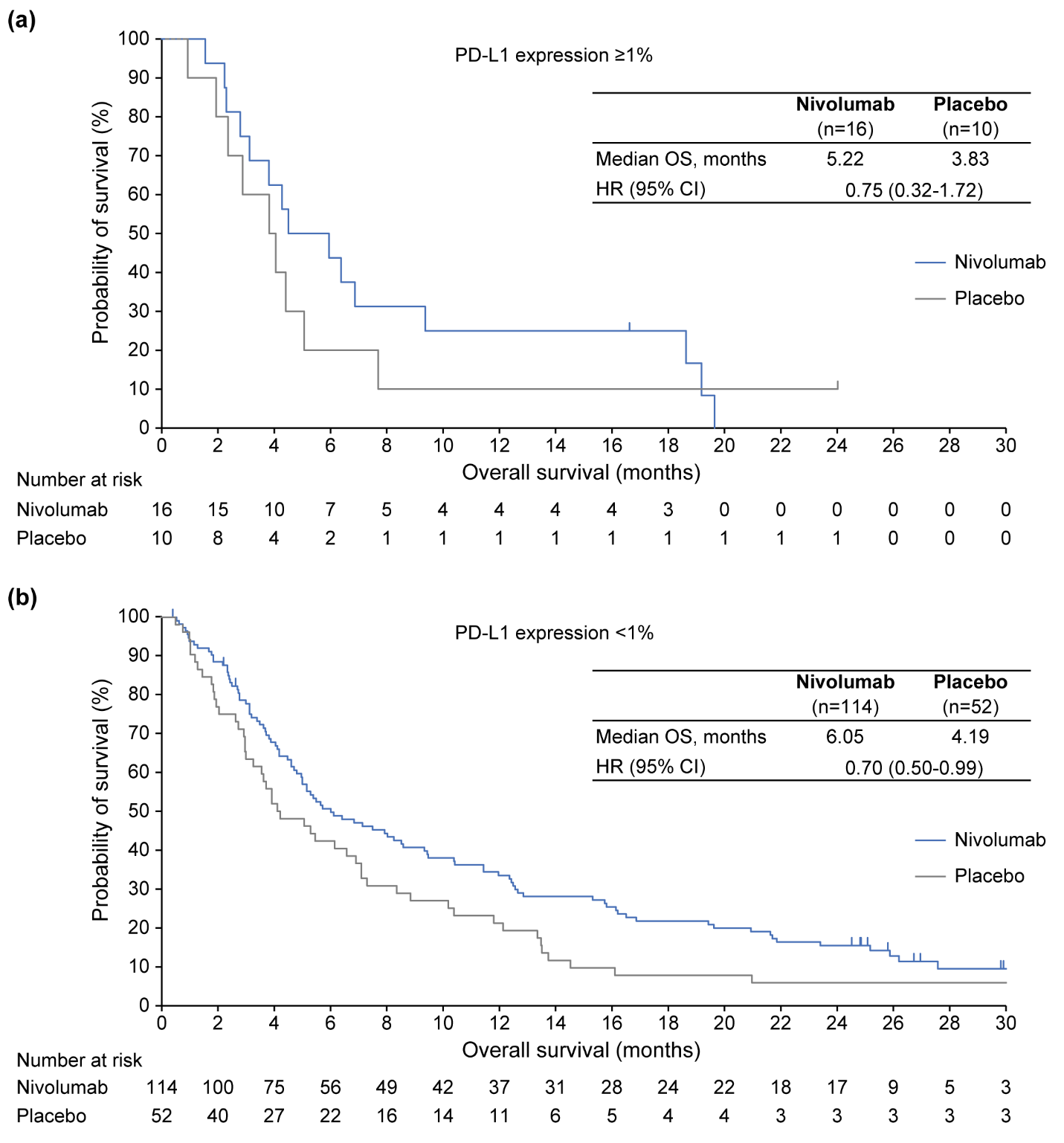


**Online Resource Fig. 3**


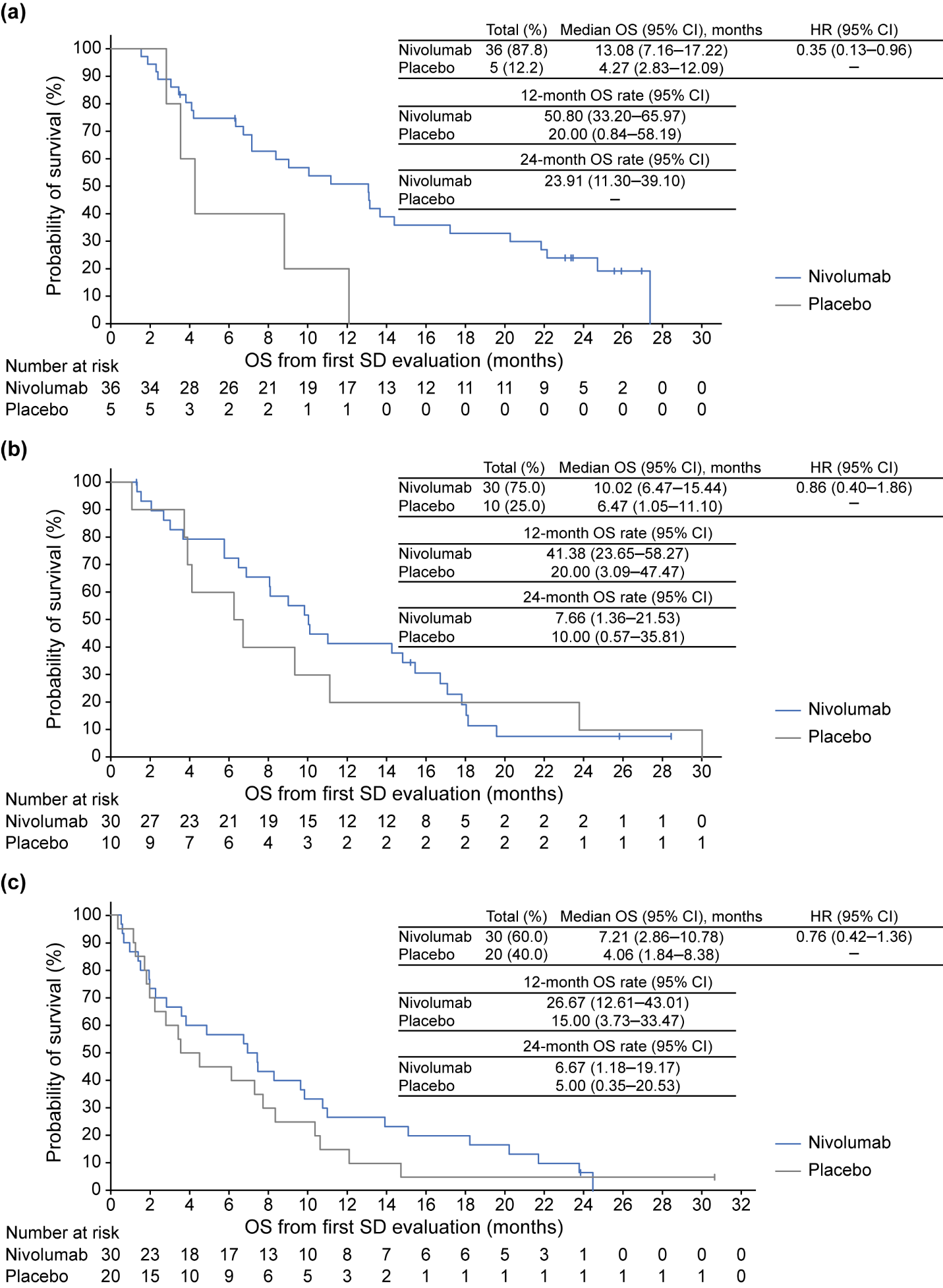

Supplement: Supplementary file 1 — Supplementary file1 (DOCX 1539 kb) [file 10120_2019_1034_MOESM1_ESM.docx]
